# BaSO_4_/TiO_2_ Microparticle Embedded in Polyvinylidene Fluoride-Co-Hexafluoropropylene/Polytetrafluoroethylene Polymer Film for Daytime Radiative Cooling

**DOI:** 10.3390/polym15193876

**Published:** 2023-09-25

**Authors:** Mohamed Mahfoodh Saleh Altamimi, Usman Saeed, Hamad Al-Turaif

**Affiliations:** Chemical and Materials Engineering Department, Faculty of Engineering, King Abdulaziz University, Jeddah P.O. Box 80200, Saudi Arabia; ms0039@stu.kau.edu.sa (M.M.S.A.); hturaif@kau.edu.sa (H.A.-T.)

**Keywords:** daytime radiative cooling, BaSO_4_, TiO_2_, PVDF, PTFE, solar reflectance

## Abstract

Radiative cooling is a new large-scale cooling technology with the promise of lowering costs and decreasing global warning. Currently, daytime radiative cooling is achieved via the application of reflective metal layers and complicated multilayer structures, limiting its application on a massive scale. In our research, we explored and tested the daytime subambient cooling effect with the help of single-layer films consisting of BaSO_4_, TiO_2_, and BaSO_4_/TiO_2_ microparticles embedded in PVDF/PTFE polymers. The film, consisting of BaSO_4_/TiO_2_ microparticles, offers a low solar absorbance and high atmospheric window emissivity. The solar reflectance is enhanced by micropores in the PVDF/PTFE polymers, without any significant influence on the thermal emissivity. The BaSO_4_/TiO_2_/PVDF/PTFE microparticle film attains 0.97 solar reflectance and 0.95 high sky-window emissivity when the broadly distributed pore size reaches 180 nm. Our field test demonstrated that the single-layer BaSO_4_/TiO_2_/PVDF/PTFE microparticle film achieved a temperature 5.2 °C below the ambient temperature and accomplished a cooling power of 74 W/m^2^. Also, the results show that, when the humidity rises from 33% to 38% at 12:30 pm, it hinders the cooling of the body surface and lowers the cooling effect to 8%.

## 1. Introduction

Global warming is increasing day by day and is expected to rise significantly, by up to 0.3–4.8 °C, compared to the start of the 21st century. The annual average surface air temperature in Saudi Arabia in the past 50 years has risen by 1–2 °C [1,2]. In the middle of summer 2021, in some regions of Saudi Arabia, the temperature increased to 45 °C. Research has confirmed that the emission of greenhouse gases is the main culprit causing an increase in global warming [3,4,5]. Also, there is an increase in the use of air conditioning and other electrical devices to combat the slowly increasing, unbearable atmospheric temperatures, which elevates electricity costs and demand [6,7]. In order to make cooling cost-effective, there is an urgent need for innovative solutions and materials. One of the methods employed is passive daytime radiative cooling (PDRC), where a surface on the ground radiates heat towards the cold external space through the long-wave infrared (LWIR) atmosphere window (λ, 8 μm–13 μm) [8,9,10,11,12,13,14]. The development of an effective daytime radiative cooling film material that has the quality of appropriate solar reflectance (P_solar_) in order to reduce solar heat addition and efficient emittance (ε_LWIR_), to magnify radiative heat subtraction, is still a challenging task [15,16,17,18]. Previously, TiO_2_ films on glass were developed with lower concentrations of particles, and the performance of these films was insufficient because of the ineffective solar reflection, as the films had an increased solar absorbance in the ultraviolet range (UV) [19]. To eliminate the problem of high UV absorption, experiments with wide-bandgap materials were performed [19,20,21,22,23,24,25].Recently, the use of multilayer and photonic structure films has shown an effective daytime subambient cooling potential [18,26]. The downsides of these methods include the use of a metallic layer, an excessive thickness, and complex structures. The extreme coating thickness of the film has a significant effect because the reflected radiation increases with the increased coating thickness. Moreover, other research has demonstrated the use of a dual-layer design devoid of metal usage. In the research, solar reflectance was achieved via a TiO_2_ top layer, and thermal emission was reached via a bottom layer, resulting in partially efficient cooling [27,28]. Also, a dense single-layer film on glass was manufactured with SiO_2_ particles, which resulted in a limited daytime cooling ability [29]. Li et al. [30] suggested a BaSO_4_ microparticle film on a glass substrate, achieving a high value of solar reflectance and emissivity. Furthermore, Chae et al. [31] experimented with the large bandgaps already found in SiO_2_ and Al_2_O_3_ particle-coated films on glass, which simultaneously showed a harmonized infrared emission ability in an atmospheric window, resulting in effective radiative cooling in summertime, with a surface temperature drop of 7.9 °C in comparison to the ambient temperature. The utilization of particles in order to reflect sunlight means that the films absorb λ < 410 nm, which limits the R_solar_ to ≈0.87 in value and, in return, reduces the radiative cooling ability in the middle of the day [29]. Xue et al. [32] included fluorescent particles in the fabrication of radiative cooling films, resulting in an improved reflectivity in the radiative cooling films, covering the complete solar radiation range. Liu et al. [33] provided a super-hydrophobic silica aerogel and layer-by-layer structure, including silica aerogel and an extra phase-change material layer with a low thermal conductivity and a high contact angle. To deal with the problem of limited reflectance, experiments with porous polymers including polyvinylidene fluoride-co-hexafluoropropene (PVDF-HF) were performed, and the results showed an elimination of absorption by TiO_2_ particles [34,35]. The presence of adequate micropores in the films effectively scatters radiation from the ultraviolet to the infrared spectrum, resulting in an increased R_solar_ to 0.93–0.94 and ε_LWIR_ to 0.92–0.95. The porous polymeric film was able to acquire a subambient cooling of ≈4 °C when placed in direct sunlight [24,32]. Also, the use of polymethyl methacrylate (PMMA), polytetrafluoroethylene (PTFE), polyetherimide (PEI), and polydimethylsiloxane (PDMS) porous polymer added to the durability of films for long-standing outdoor implementation for radiative cooling films [36,37]. Cai et al. studied cellulose nanocrystal aerogel for efficient radiative cooling, as it provides the ultrahigh solar reflectance of 97.4% and the high infrared emittance of 94% [38]. Song et al. proposed a porous thermoplastic urethane (TPU) membrane with the high thermal emissivity of 95% and a sunlight reflectivity of 93% [39]. Xiameng et al. showed that the presence of a porous structure and a Si–O–Si chemical bond in thermoplastic polyurethane aerogels (TPUAs) resulted in a value of 0.95 for solar reflectance and emissivity, which, in the end, achieved a subambient cooling temperature of 10.6 °C with respect to the surrounding temperature [40,41]. The effectiveness of cooling coatings during the daytime, especially around noon, depends on the combating of two factors, which are solar irradiation and exposure to direct sunlight. These environmental factors can reduce the effectiveness of coatings because a higher cooling power of up to 1000 W/m^2^ will be required. In this study, we explored full summer daytime subambient radiative cooling, with a final product made of BaSO_4_- and TiO_2_-embedded particles in a PVDF/PTFE matrix, using the spin-coating method. We selected BaSO_4_ and TiO_2_ because they are cost-effective and have a high electron band, which proves to be useful for lower phonon resonance and solar absorption. By implementing a useful particle size and an ample particle size distribution in a BaSO_4_/TiO_2_ microparticle film, we were able to achieve the high window emissivity of about 0.95 and the high solar reflectance of about 0.97. The field test showed that the film achieved a surface temperature of less than 4.5 °C below the ambient temperature. To ensure the proper functioning of the radiative coating, 30 wt.% concentrations of BaSO_4_ and TiO_2_ were embedded in the PVDF/PTFE matrix. The 30 wt.% concentrations of BaSO_4_ and TiO_2_ microparticles were added to control the low refractive index of TiO_2_ and BaSO_4_. In the research presented, we explored and fabricated BaSO_4_/TiO_2_/PVDF/PTFE films using spin coating. A strategy was developed which can control the absorptivity spectra of the emitter, which will help in creating effective sub-ambient cooling during the daytime by minimizing the absorbed heat from the environment (*P*atm and *P*sun) and by maximizing the heat emitted from the emitter (*P*rad). We emphasized the obtention of readings and results over midday, from 10:30 to 14:30. We intended to observe whether the radiative cooling coating was capable of achieving 5–8 °C subambient temperatures, could also reflect about 97% sunlight, and could emit LWIR infrared radiation. The films showed an efficient R_solar_ (0.97) and ε_8–13μm_ (0.95). Additionally, with a solar intensity of about ~900 W/m^2^, the films also demonstrated subambient cooling of 6.1 °C and a cooling power of ~74 W/m^2^. Moreover, with a humidity of 38% and a solar intensity of ~900 W/m^2^, the films showed subambient cooling of ~4.5 °C. The results of the experiment verified that the implementation of the BaSO_4_/TiO_2_/PVDF/PTFE films not only increased solar reflectance but also enhanced thermal emittance.

## 2. Materials and Methods

### 2.1. Theoretical Model of Radiative Cooling Performance

**Figure 1:**
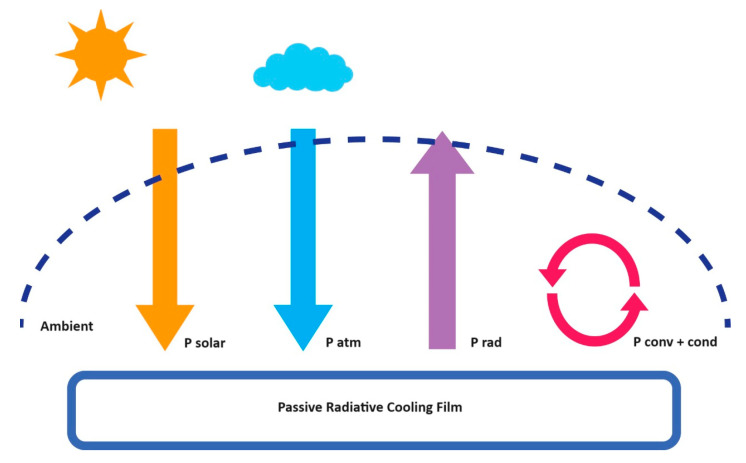
Schematic representation of the cooling processes on the material surface. Solar radiation: yellow, Atmospheric radiation: blue, Power radiation: purple, power reduction: red.

The radiative cooling films, in daylight, are exposed to solar irradiance and atmospheric thermal radiation related to the ambient air temperature (*T_amb_*) at the same time [15]. Figure 1 and Equation (1) show the net cooling process *P_cool_* of radiative cooling.

(1) $$P_{cool}\left(T\right)=P_{rad}\left(T\right)-P_{atm}\;(T_{amb})\;-P_{sun-}P_{cond+conv}$$

In Equation (1), the power radiated outward by the film is presented by Equation (2):

(2) $$P_{rad}\left(T\right)=A\int d\Omega Cos\theta \int_{0}^{⋈}d\lambda I_{BB}\left(T,\lambda \right)\varepsilon \left(\lambda ,\theta \right)$$

$\int d\lambda =2\pi \int_{0}^{\frac{\pi }{2}}d\theta Sin\theta$ is the angular integral over a hemisphere, $I_{BB}\left(T,\lambda \right)=\frac{2hC^{2}}{\lambda^{5}}\frac{1}{e^{hCiK_{B}T}-1}$ is the blackbody spectral radiance at temperature T (K), where k_B_ is the Boltzmann constant (J/K), c is the speed of light (m/s), h is Planck’s constant (J-s), and λ is the wavelength.

(3) $${\;\;\;\;\;\;\;\;\;\;P}_{atm}\left(T_{amb}\right)=A\int d\Omega Cos\theta \int_{0}^{⋈}d\lambda I_{BB}\left(T_{amb},\lambda \right)\epsilon \left(\lambda ,\theta \right)\epsilon_{atm}\left(\lambda ,\theta \right)$$

Equation (3) shows the absorbed power due to the incident atmospheric thermal radiation.

(4) $$P_{Sun}=A\int_{0}^{⋈}d\lambda \epsilon (\lambda ,\theta_{Sun}I_{AM1.5}\left(\lambda \right)$$

Equation (4) presents the incident solar power absorbed by the structure. We reached Equations (3) and (4) by implementing Kirchhoff’s radiation law to substitute the absorptivity with the emissivity (λ, θ). The emissivity of the atmosphere is presented by ε_atm_ (λ, θ)= 1− t(λ) ^1/c^°^s θ^, where t(λ) shows the atmospheric transmittance in the zenith direction [35,36]. In Equation (4), the solar illumination is indicated by I_AM1.5_ (λ), which is the AM1.5 spectrum. We supposed that the experimental fabricated structure was in front of the sun at a set angle θ_Sun_. This means that the P_Sun_ does not have an angular integral, and the cooling emissivity is represented by its value at θ_Sun._

(5) $$P_{cond+conv}\left(T,T_{amb}\right)=Ah_{c}\left(T_{amb}-T\right)$$

Equation (5) presents the power reduction due to conduction and convection. This means h_c_ = h_cond_ + h_conv_ is a merged nonradiative heat coefficient that attains the combined effect of convective and conductive heating due to the correlation of the radiative cooling films surfaces with the air near the radiative cooling films.

### 2.2. Materials and Cooling Setup

**Figure 2:**
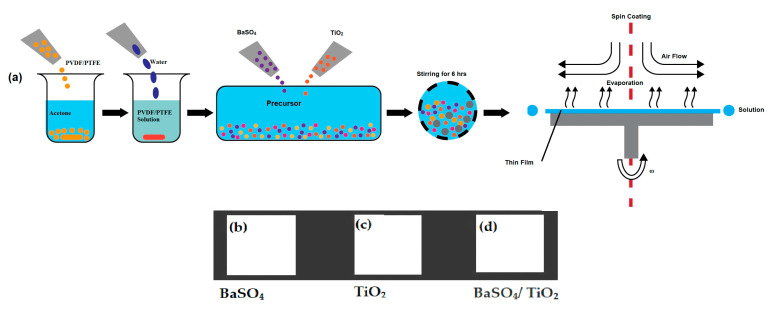
(**a**) Schematic illustration of the synthetic process: (**b**) BaSO_4_ film; (**c**) TiO_2_ film; (**d**) BaSO_4_/TiO_2_ film.

Polyvinylidene fluoride-co-hexafluoropropylene (PVDF-HF-99%), polytetrafluoroethylene (PTFE-99%), barium sulfate (BaSO_4_ 97%), and titanium oxide (TiO_2_ Rutile99.8%) were purchased from Alfa Aesar GMBH (Karlsruhe-Germany). The particle size of BaSO_4_ is 1–1.4 microns, while that of TiO_2_ is 0.9–1.6 microns. First, 1 g of PVDF HF and 1 g of PTFE were dissolved in 5 g acetone to create the PVDF-HFP/PTFE solution. Then, 3 g of deionized water was mixed with the solution to make the PVDF-HFP/PTFE–acetone–water precursor solution. Also, microparticles were included to achieve a weight percentage of 30% for BaSO_4_/TiO_2_ in the PVDF/PTFE polymer blend solution. Following stirring for 6 h, the microparticles were randomly dispersed into the PVDF/PTFE polymer blend. Finally, the BaSO_4_/TiO_2_/PVDF/PTFE solution was utilized to develop film on a 3 mm thick glass plate via spin coating with a speed of 750 rpm at 25 °C and under a vacuum of 0.8 bar. The deposited film on the glass substrate was placed inside the furnace at 60 °C and cured for one hour. Additionally, the same method was used for the preparation of BaSO_4_ and TiO_2_ in the PVDF/PTFE blend solution. The schematic illustration of the synthetic process of film fabrication is presented in Figure 2.

**Figure 3:**
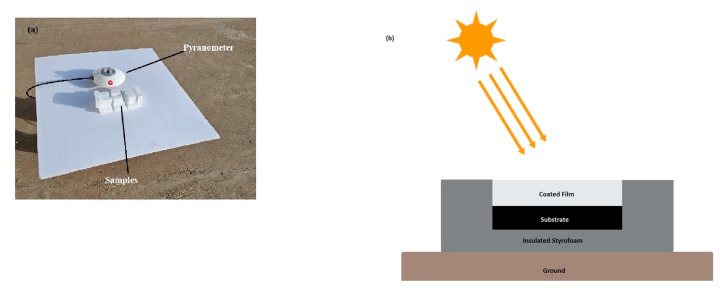
(**a**) Photograph of the setup for the outdoor surface cooling measurement in Jeddah, Saudi Arabia. (**b**) Schematic side view of the outdoor radiative film.

The radiative cooling film of 50 μm deposited on a 3 mm thick glass plate was placed on insulated foam in direct sunlight, as shown in Figure 3a. All three samples were placed together to allow for comparison of the results. Also, the experimental setup was raised 10 cm above the ground to avoid any heat conduction from the ground. The design of the setup is shown schematically in Figure 3b. The model used, mentioned in Section 2.2, for the losses of conduction and convection of the experimental setup to the BaSO_4_/TiO_2_/PVDF/PTFE radiative cooling film utilized the h_c_ value of 6.9 W/m^2^ K. The heat input to the outdoor environment mainly resulted from solar radiation [11].

### 2.3. Characterization

A scanning electron microscope (JEOL, Tokyo, Japan—JSM-7600F) was employed to examine the surface features of the radiative cooling film. Also, an energy dispersive X-ray (EDS) test was implemented to assess the variety of elements typically found on the surface of the film. Raman spectroscopy (Thermo Fisher Scientific, Waltham, MA, USA–DXR Raman Microscope) was used to recognize the components of the film. A UV-Vis-NIR spectrometer (PerkinElmer, Hong Kong, China—Lambda 750) was utilized to determine the reflectance spectrum in the 0.3–2.5 µm wavelength. The absorptivity and emissivity in the wavelength range of 2.5–16 µm were measured using a Fourier transform infrared spectrometer (Thermo Fisher Scientific—Nicolet iS10) under ATR mode. Rooftop measurements were performed on 10 May 2023 and 16 May 2023 in Jeddah, Saudi Arabia under a clear sky. The solar irradiance was determined using an SR25 secondary standard pyranometer (Huskflex Thermal Sensors, Delft, The Netherlands) with a sapphire outer dome and an L119 data logger. A laser IR thermometer was provided to evaluate the surface temperature of the film at intervals of 30 min. Moreover, a temperature detector was placed beside the setup to measure the ambient temperature surrounding the film. The humidity was taken from the Saudi weather network [42].

## 3. Results and Discussion

**Figure 4:**
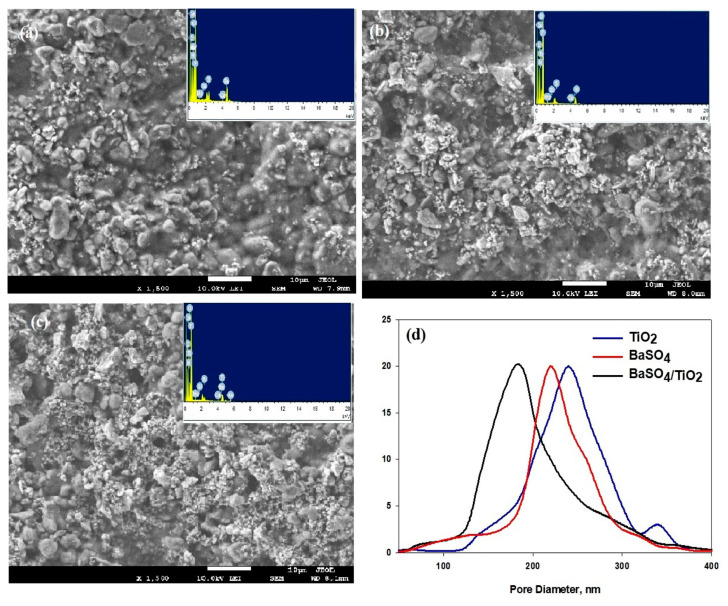
SEM micrographs of the coatings with the EDS results. (**a**) BaSO_4_ specimen; (**b**) TiO_2_ specimen; (**c**) BaSO_4_/TiO_2_ specimen; (**d**) pore size distribution in the TiO_2_, BaSO_4_, and BaSO_4_/TiO_2_ films.

The SEM micrographs of the BaSO_4_/PVDF/PTFE, TiO_2_/PVDF/PTFE, and BaSO_4_/TiO_2_/PVDF/PTFE films are presented in Figure 4a–c. The micrograph demonstrates that the BaSO_4_ and TiO_2_ microparticles were elongated, rod-shaped, and randomly distributed in the developed three films. The EDS results showed the presence of C, F, and O, which resulted from the coated film particles in the structure of the PVDF and PTFE polymers’ matrix. Additionally, the presence of other particles was confirmed, including Ba and Ti, which are related to the embedded particles in the TiO_2_/PVDF/PTFE, BaSO_4_/PVDF/PTFE and TiO_2_/BaSO_4_/PVDF/PTFE films in weight%. Table 1 presents the elemental composition of the films obtained via EDS analysis. The image J software (https://imagej.nih.gov/) was used to analyze the pore size. An average pore size of 180 nm was determined for the TiO_2_/BaSO_4_ film, which was 10–15% smaller than the TiO_2_ (250 nm) and BaSO_4_ (230 nm) films, as described in Figure 4d.

**Figure 5:**
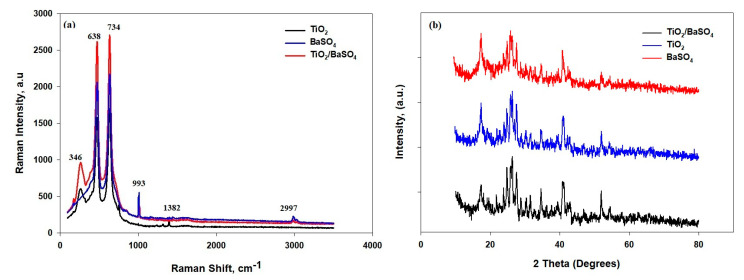
(**a**) Raman spectra of the BaSO_4_, TiO_2_, and BaSO_4_/TiO_2_films; (**b**) the XRD pattern of the BaSO_4_, TiO_2_, and BaSO_4_/TiO_2_ films.

Figure 5a was used to determine the components of the film via Raman spectroscopy. The peak at 638 cm^−1^ is associated with CH_2_ rocking, which is related to the α and β phases of PVDF-HF. The peak at 2997 cm^−1^ is associated with CH_2_ symmetric stretching and is related to the β phase of PVDF-HF [18]. The peaks at 471 cm^−1^ are related to the torsional and deformation vibrations of CF_2_. The peak at 734 cm^−1^ is related to symmetric CF_2_ stretching, while the 1382 cm^−1^ peak is related to C-C stretching. All these peaks are directly related to PTFE. The rutile TiO_2_ shows peaks at 346 cm^−1^, which correspond to antisymmetric bending vibrations of O–Ti–O in the TiO_2_ microparticles. The O–Ti–O is presented in the TiO_2_ and TiO_2_/BaSO_4_ microparticle films. The peak at 993 is associated with the symmetric stretching mode of the S–O bond in BaSO_4_, which is demonstrated in the BaSO_4_ and TiO_2_/BaSO_4_ films.

The XRD pattern of the BaSO_4_/PVDF/PTFE, TiO_2_//PVDF/PTFE, and BaSO_4_/ TiO_2_/PVDF/PTFE films is shown in Figure 5b. Most of the peaks are overlapping because of the presence of the PVDF and PTFE polymer matrix with embedded particles of TiO_2_ and BaSO_4_ in the developed film. Figure 5b also demonstrates the distinct peaks found at the diffraction angles of 18.13°, 19.675°, and 26.93° corresponding to the kinetically stable α-phase of PVDF. The PTFE of every film shows intensity at the angles of 18.25°, 31.80°, and 36.85°. The peaks in PVDF and PTFE can be noticed in every diffraction pattern of the three films due to their presence in the matrix. The diffraction peaks corroborate the presence of the rutile phase of TiO_2_ at angles of 25.23°, 37.71°, 47.72°, 54.16°, 55.32°, and 62.54° with crystal planes of (101), (004), (200), (105), (211), and (204), respectively. Furthermore, the peaks appear at 19.99°, 20.46°, 22.80°, 24.87°, 25.86°, 26.8.5°, 28.76°, and 31.62°. These peaks correspond to the orthorhombic BaSO_4_ crystal planes of (200), (011), (111), (002), (210), (120), (211), and (112), respectively, which can be noticed in the BaSO_4_ and TiO_2_/BaSO_4_ films. Finally, the developed films are composed of elements that are essential for the intended function of passive radiative cooling.

**Figure 6:**
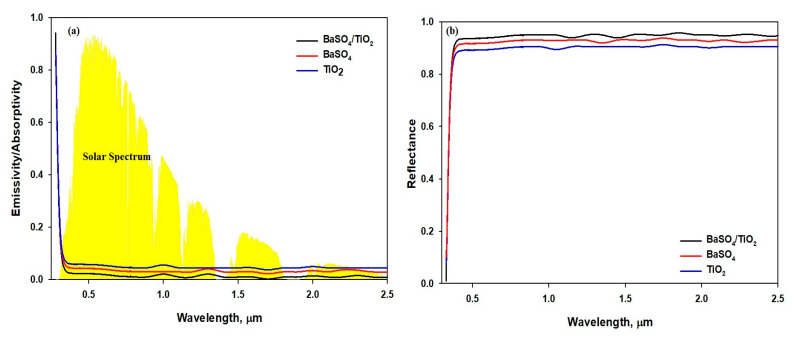
(**a**) Emissivity/absorptivity spectra across the solar wavelengths. (**b**) Reflectance spectra across the solar wavelengths.

Figure 6 shows the optical properties of the TiO_2_/PVDF/PTFE, BaSO_4_/PVDF/PTFE, and TiO_2_/BaSO_4_ /PVDF/PTFE films. As shown in Figure 6a, the TiO_2_/ BaSO_4_ /PVDF/PTFE film showed a lower UV light absorption to some extent, compared to the TiO_2_ and BaSO_4_ films. The reflectivity of the coating in the 0.3–2.5 μm region is attributed to the low absorption of solar light [43]. Also, when PVDF/PTFE was utilized as the matrix, the reflectance of the three developed films with the similar thickness of 50 μm differed slightly, which is shown by the high reflectance (R_solar_) of 0.97 of the TiO_2_/BaSO_4_/PVDF/PTFE film in comparison with the TiO_2_/BaSO_4_/PVDF/PTFE andBaSO_4_/PVDF/PTFE films, as demonstrated in Figure 6b. The pore size of 180 nm found in the TiO_2_/BaSO_4_/PVDF/PTFE film can be attributed to the high solar reflectance and low solar absorption [15]. Additionally, the BaSO_4_ microparticle has a high electronic band gap (7.27 ev), which should accomplish low solar absorption; meanwhile, the pore size of the BaSO_4_/PVDF/PTFE film is 230 nm. Similarly, the TiO_2_ microparticles have a low electronic band gap (3.2 ev) and the higher pore size of 250 nm, which provide a high absorption and low reflectivity. We determined that a high solar reflectance (R_solar_) can be attained through employing the porous structure of the film. The submicron pores scatter sunlight and direct the back scattering effect [15,44]. The pore size of 180 nm found in the TiO_2_/BaSO_4_/PVDF/PTFE film might reduce the scattering path and the transmission, improving the scattering of a shorter visible wavelength.

**Figure 7:**
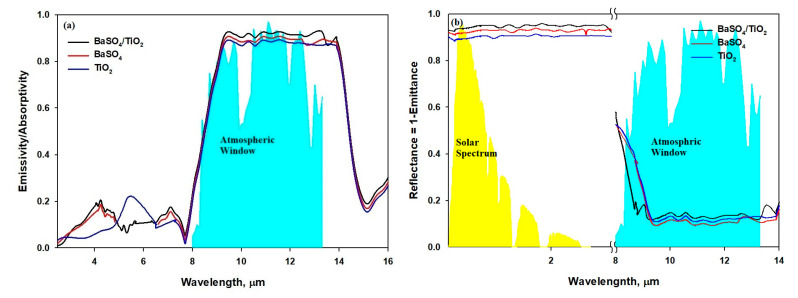
(**a**) Emissivity/absorptivity spectra of the BaSO_4_/TiO_2-_microparticle-embedded films. (**b**) Reflectance spectra through the atmosphere of solar energy at 0.3–2.5 microns and the atmospheric window from 8 to 13 microns.

Figure 7a demonstrates that the three films show a high mid-infrared emissivity as selective or broadband emitters, even though the matrix of the film is PVDF/PTFE. Also, more sunlight is reflected, as the particles enhance the scattering of sunlight. The study shows that an excellent absorbent film is also an excellent radiator, which, in return, improves the emissivity potential [43]. Figure 7b shows that almost all solar energy reaches the film surface in the range from 0.3μm to 2.5 μm, and the surface cooling radiation is in LWIR, which is focused generally in the atmospheric window from 8μm to 13 μm. The difference can be utilized through developing materials that can reflect radiation in the range from 0.3 μm to 2.5 μm and absorb minimum radiation, while emitting radiation in the 8 μm-to-13 μm range [18,26]. We found that the developed film is capable of strong reflectance in the range of 0.3–2μm and selective emissivity towards the atmosphere window in the range from 8 μm to 13 μm. The average emissivity (ε_8–13μm_) of the BaSO_4_/TiO_2/_PVDF/PTFE film was around 95% falling in the range of 8–13 μm. The emissivity of the developed TiO_2/_PVDF/PTFE, BaSO_4_/TiO_2/_PVDF/PTFE, and BaSO_4_/TiO_2/_PVDF/PTFE films did not differ significantly. The 97% reflectivity (R_solar_) of the BaSO_4_/TiO_2/_PVDF/PTFE film occurred in the range of 0.3–2.5 μm, which is 8–10% more than the reflectivity of the TiO_2/_PVDF/PTFE and BaSO_4/_PVDF/PTFE films. The study shows that the BaSO_4_/TiO_2/_PVDF/PTFE film not only has a high reflectivity (R_solar_) but also has a good emissivity (ε_8–13μm_), resulting in a suitable cooling ability.

**Figure 8:**
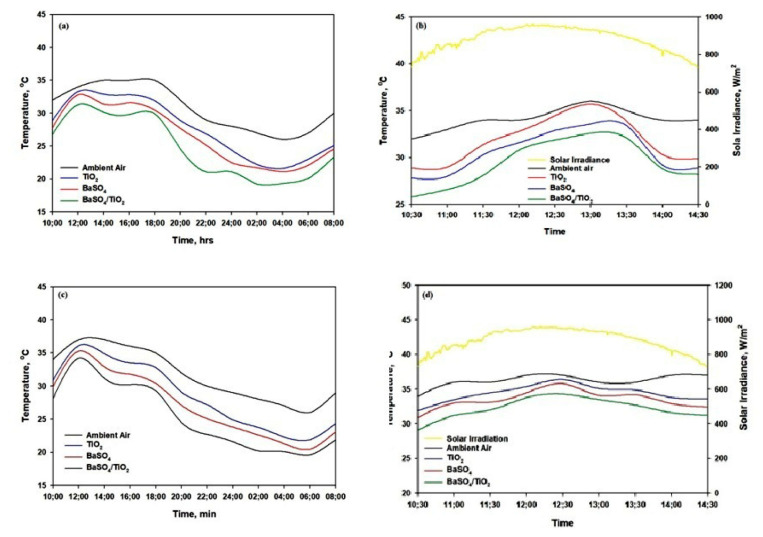
(**a**) Temperatures of the BaSO_4_, TiO_2_, and BaSO_4_/TiO_2_ films, measured on 10 May 2023. (**b**) Daytime temperature of the BaSO_4_, TiO_2_, and BaSO_4_/TiO_2_ films, measured on 10 May 2023. (**c**) Temperatures of the BaSO_4_, TiO_2_, and BaSO_4_/TiO_2_ films, measured on 16 May 2023. (**d**) Daytime temperature of the BaSO_4_, TiO_2_, and BaSO_4_/TiO_2_ films, measured on 16 May 2023.

Figure 8 shows the performance of the radiative film under direct sunlight during the summer days of 10 May 2023 and 16 May 2023 in Jeddah, Saudi Arabia. As shown in Figure 8a, the BaSO_4_/PVDF/PTFE and TiO_2/_PVDF/PTFE films accomplished subambient cooling of about 4–5 °C during the night but maintained the same temperature as the ambient environment over midday. On the other hand, the BaSO_4_/TiO_2/_PVDF/PTFE film demonstrated excellent radiative cooling during both nighttime and daytime. The average below-ambient temperature of the BaSO_4_/TiO_2/_PVDF/PTFE is ~8 °C during the night (between 18:00 and 4:00) and ~5.7 °C over midday (between 10:30 and 14:30). The effectiveness of the ambient cooling material on hot summer days depends on two important factors, which are a high solar reflectance (Rsolar) and high emissivity [40,41]. The solar reflectance (R_solar_ ≈ 1) should occur between the wavelength range of 0.2 and 2.5 μm to prevent solar absorption. The value of emissivity (ε_LWIR_ ≈ 1) should lie in the range of the long-wavelength infrared (LWIR) atmospheric transparency window from 8 to 13 μm to efficiently reflect heat into cold space. The evaluation of the peak daytime solar radiation intensity was conducted on 10 May 2023 between 10:30 and 14:30. The solar radiation intensity during the selected time period was highest during the day, and the maximum value recorded was about 900–950 W m^2^. The daytime radiative cooling results presented in Figure 8b show that the ambient temperature increased to about 36 °C. The PVDF/PTFE films exhibited lower temperatures than the ambient temperature, and the temperature drop was proportional to the *R*solar. The average temperature drops (10:50–12:50: sunlight intensity = 950 W m/^2^) of the BaSO_4_/TiO_2/_PVDF/PTFE, BaSO_4_/TiO_2/_PVDF/PTFE, and TiO_2/_PVDF/PTFE were 5.7, 3.4, and 1.2 °C, respectively. Xiameng et al. found similar behavior by considering the highest solar intensity (800 w/m^2^) in the midday from 9:30 to 15:30 for thermoplastic polyurethane (TPU) films [41]. Figure 8c,d present the performance of the radiative film during the summer day of 16 May 2023 in Jeddah, Saudi Arabia. The BaSO_4_/PVDF/PTFE and TiO_2/_PVDF/PTFE films were able to achieve subambient cooling of about 2–4 °C during the night but also maintained a similar temperature to the ambient temperature over midday. The mean cooling temperature below the ambient temperature of the BaSO_4_/TiO_2/_PVDF/PTFE was ~7.5 °C during the night (between 18:00 and 6:00) and ~4.5 °C over midday (between 10:30 and 14:30). As shown in Figure 8d, with regard to the humidity at 10:30 local time, the temperature drop for the BaSO_4_/TiO_2/_PVDF/PTFE film was 4.8 °C compared to the ambient temperature, even though a noticeable solar irradiance of 880 W/m^2^ was directed onto the film. The film maintained a temperature 2.3–2.5 °C below the ambient temperature between 11:30 and 13:30, when the solar irradiance was 900–920 W/m^2^. Moreover, the BaSO_4/_PVDF/PTFE film reached 35 °C, which was 1.2 °C below the ambient temperature, and the TiO_2/_PVDF/PTFE film reached 36 ° C, which was 0.5 °C below the ambient temperature. We found that changes in the humidity from ~33% to ~38% at 12:30 affected the film cooling temperature and reduced it to about ~4–2 °C, as shown in Figure 8d. The optimum performance of the cooling films depends on the geographical location and weather of the place where it will be employed [18,45,46]. The BaSO_4_/TiO_2_/PVDF/PTFE film demonstrated a high Rsolar, and the heat gain was significantly reduced. Furthermore, the high emissivity of the BaSO_4_/TiO_2_/PVDF/PTFE films overlapped with the atmospheric transparency window through which heat efficiently radiated towards extremely cold outer space. Finally, the outcomes of the study indicate that the BaSO_4_/TiO_2/_PVDF/PTFE film is the most efficient daytime radiative cooling material when exposed to extremely hot weather conditions. The film, with a high solar reflectance and emissivity that can greatly minimize solar absorbance and thermal radiation emission, will be suitable in the climate found in Saudi Arabia.

**Figure 9:**
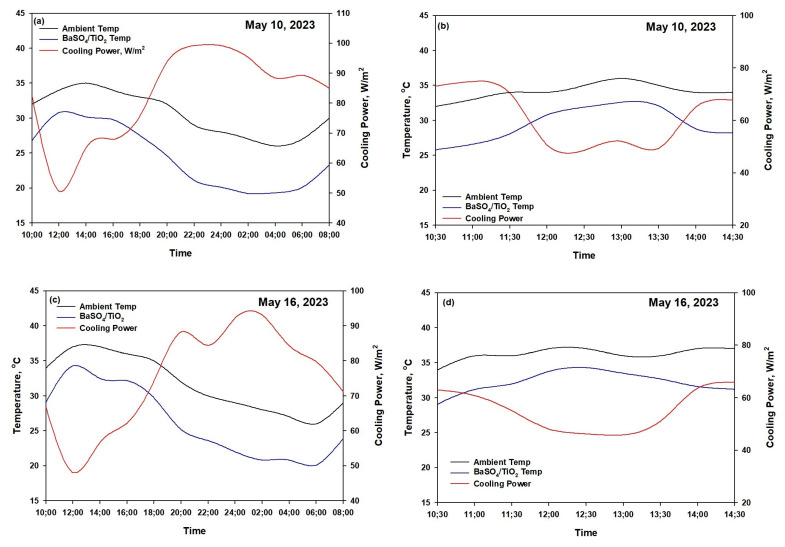
(**a**) Temperature and cooling power during the day and night for the BaSO_4_/TiO_2_ film on 10 May 2023. (**b**) Temperature and cooling power from 10:30 to 14:30 for the BaSO_4_/TiO_2_ film on 10 May 2023. (**c**) Temperature and cooling power during the day and night for the BaSO_4_/TiO_2_ film on 16 May 2023. (**d**) Temperature and cooling power from 10:30 to 14:30 for the BaSO_4_/TiO_2_ film on 16 May 2023.

The BaSO_4_/TiO_2/_PVDF/PTFE film shows significant potential for radiative cooling for buildings due to its high solar reflectance. Due to the results for the BaSO_4_/TiO_2/_PVDF/PTFE film, the cooling power was measured using the net cooling equations, as mentioned in the theoretical modeling section, with an h_c_ value of 6.9 W/m^2^ K and the temperature measurement. Figure 9 shows the cooling ability of the BaSO_4_/TiO_2/_PVDF/PTFE film, measured via considering the drops in temperature. The mean below-ambient temperature of the BaSO_4_/TiO_2_/PVDF/PTFE film is ~8 °C during the night (between 18 p.m. and 6 a.m.) and ~5.7 °C over midday (between 10:00 and 14:30). The onsite measurement of the cooling capability in Jeddah on 10 May 2023 demonstrated that the cooling power reached an average of 88 W/m^2^ during the night and 75 W/m^2^ during the day, as shown in Figure 9a. Figure 9b presents the cooling capability of the BaSO_4_/TiO_2/_PVDF/PTFE films, which reached about 74 W/m^2^ at 10:30 am, with a temperature drop, ΔT (T_ambient_ − T_surface_), of 6.2 °C, with a humidity of 33%. Also, Figure 9b shows that the cooling power was 50 W/m^2^ at 12:30, with aΔT of 3.9 °C. The results show that the average cooling power over four hours is 63.5 W/m^2^, with a humidity of 33%. Similarly, the day- and nighttime cooling power on 16 May 2023 was observed as shown in Figure 9c. The average below-ambient temperature of the BaSO_4_/TiO_2/_PVDF/PTFE was ~7.5 °C during the night (between 18:00 and 6:00) and ~4 °C over midday (between 10:00 and 14:30). The measurement of the cooling power in Jeddah on 16 May 2023 showed that the cooling power reached an average of 83.2 W/m^2^ during the night and 58 W/m^2^ during the day. It was found that the cooling power was 65 W/m^2^ at 10:30, with a ΔT of 4.9 °C and a solar radiance of 730 W/m^2^ and, eventually, the cooling power was 48 W/m^2^ at 12:30, with a ΔT of 2.1 °C and a solar radiance over 940 W/m^2^, as shown in Figure 9d. The obtained data demonstrate that the average cooling power over four hours was 55.5 W/m^2^ with 38% humidity. The thermal emission increased with a high surface temperature over midday, from 11:30 to 13:30, which balanced the higher solar absorption. Finally, we concluded that when the humidity increased from 33% to 38% at 12:30, the temperature drop of the film decreased from ~4 °C to ~2 °C, which, in turn, also lowered the cooling power of the BaSO_4_/TiO_2_/PVDF/PTFE film by about 8%.

## 4. Conclusions

The optical properties of the BaSO_4_/TiO_2_, BaSO_4_, and TiO_2_ films were examined for their suitability in a radiative cooling application developed via the method of spin coating. The thickness of the film is 50 μm, and the weight percentage of the TiO_2_, BaSO_4_, and BaSO_4_/TiO_2_ microparticles in the PVDF/PTFE matrix is 30%. The BaSO_4_/TiO_2_/PVDF/PTFE film showed the most suitable results in comparison with the BaSO_4_/PVDF/PTFE and TiO_2_/PVDF/PTFE films. The emissivity (ε_8–13μm_) and solar reflectance (R_solar_) of the BaSO_4_/TiO_2_/PVDF/PTFE film were 0.95 and 0.97, respectively. The onsite measurement of the cooling capability in Jeddah on 10 May 2023, showed that the cooling power reached an average of 88 W/m^2^ during the night on May 10, 2023. Similarly, the measurement of the cooling power in Jeddah on 16 May 2023, showed that the cooling power reached an average of 83.2 W/m^2^ during the night. The BaSO_4_/TiO_2_ film, under direct sunlight, was able to reduce the surface temperature by about 6–4 °C compared to the ambient temperature. Also, the obtained average radiative cooling power for the BaSO_4_/TiO_2/_PVDF/PTFE film on a warm summer day was about 50 W/m^2^ with a humidity of 33%, and 46.5 W/m^2^ with 38% humidity at 12:30. Finally, the results demonstrate that the BaSO_4_/TiO_2/_PVDF/PTFE film is capable of effective radiative cooling and is suitable for outdoor applications under direct sunlight, making the process of cooling cost-effective and energy-efficient.

## Figures and Tables

**Table 1:** Elemental composition of the TiO_2_//PVDF/PTFE and BaSO_4_/ TiO_2_/PVDF/PTFE films.

Elements	BaSO_4_ Film	TiO_2_ Film	BaSO_4_/TiO_2_ Film
C _k_	14.61	20.86	18.59
N _K_	0.00	0.00	0.00
O _K_	16.64	10.87	7.74
F _K_	44.04	51.51	50.24
Mg _K_	0.00	0.00	0.00
Si _K_	0.00	0.00	0.25
S _K_	8.75	0.72	3.68
Ca _K_	0.00	0.00	0.00
Ti _K_	0.00	16.04	10.44
Ba _L_	15.96	0.00	9.06

## Data Availability

Not applicable.
